# Study of Kyasanur forest disease viremia, antibody kinetics, and virus infection in target organs of *Macaca radiata*

**DOI:** 10.1038/s41598-020-67599-x

**Published:** 2020-07-28

**Authors:** Dilip R. Patil, Pragya D. Yadav, Anita Shete, Gouri Chaubal, Sreelekshmy Mohandas, Rima R. Sahay, Rajlaxmi Jain, Chandrashekhar Mote, Sandeep Kumar, Himanshu Kaushal, Pravin Kore, Savita Patil, Triparna Majumdar, Siddharam Fulari, Annasaheb Suryawanshi, Manoj Kadam, Prachi G. Pardeshi, Rajen Lakra, Prasad Sarkale, Devendra T. Mourya

**Affiliations:** 1 Indian Council of Medical Research-National Institute of Virology, Pune, Maharashtra India; 2 Department of Veterinary Pathology, Krantisinh Nana Patil College of Veterinary Science, Shirwal, Maharashtra India

**Keywords:** Microbiology, Pathogenesis

## Abstract

The present manuscript deals with experimental infections of bonnet macaques (*Macaca radiata*) to study disease progression for better insights into the Kyasanur Forest Disease (KFD) pathogenesis and transmission. Experimentally, 10 monkeys were inoculated with KFD virus (KFDV) (high or low dose) and were regularly monitored and sampled for various body fluids and tissues at preset time points. We found that only 2 out of the 10 animals showed marked clinical signs becoming moribund, both in the low dose group, even though viremia, virus shedding in the secretions and excretions were evident in all inoculated monkeys. Anti-KFDV immunoglobulin (Ig)M antibody response was observed around a week after inoculation and anti-KFDV IgG antibody response after two weeks. Anaemia, leucopenia, thrombocytopenia, monocytosis, increase in average clotting time, and reduction in the serum protein levels were evident. The virus could be re-isolated from the skin during the viremic period. The persistence of viral RNA in the gastrointestinal tract and lymph nodes was seen up to 53 and 81 days respectively. Neuro-invasion was observed only in moribund macaques. Re-challenge with the virus after 21 days of initial inoculation in a monkey did not result in virus shedding or immune response boosting.

## Introduction

Kyasanur Forest Disease (KFD) was first discovered in Sagar and Sohrab taluks of Shimoga district of Karnataka state in 1957^1^. The disease, which was endemic to Karnataka state, India, were reported from neighbouring states along the Western Ghats like Tamil Nadu, Goa, Maharashtra and Kerala in the last decade^2^. Kyasanur forest disease virus (KFDV) belongs to the tick borne encephalitis serogroup of the *Flaviviridae* family, which causes disease in humans and monkeys^3–5^. Studies revealed ticks of the species *Haemaphysalis* as the principal vector and reservoir for the virus^6^.

The disease in humans is characterized by fever, headache, myalgia, conjunctivitis, diarrhoea, vomiting, and haemorrhagic manifestations with a case fatality rate of up to 4%^7–9^. *Presbytus entellus* (Black faced langur) and *Macaca radiata* (Bonnet macaque), which are abundant in the Western Ghats region of India, are the most affected species of animals; acting as sentinels for the virus spread in an area^10,11^. The susceptibility of both the species has been proven experimentally too^11–13^. Though KFDV was isolated in 1957, the disease remained understudied due to lack of biocontainment facilities in India until recently.

Although rodent models were used for KFD study in the past, experimentally induced disease in those differed from published descriptions of human disease (mice developed neurologic disease and did not become febrile and lacked marked spleen and liver pathology) making rodent models less predictive of human KFD^14–16^. The literature available to date about KFD in *Presbytus entellus* and *Macaca radiata* is based on naturally infected dead animals or experimental infections wherein high dose of an early isolate of virus maintained by suckling mouse brain passages were used^11–13^. A decade long study conducted on monkey mortality in KFD endemic area revealed that, out of 1,046 deaths, 860 were *P. entellus* and only 186 were *M. radiata* with virus isolation percentage of 50% and 18.05% in necropsied animals respectively^17^. In agreement with these findings, an experimental infection studies conducted at Virus Research Centre, Pune between 1958 and 1970 found langurs to be highly susceptible to KFDV with per acute course of the disease compared to bonnet macaques. In bonnet macaques disease course was comparatively prolonged with few deaths during viremic phase and few during third week, with virus recovery from the brain similar to human biphasic disease wherein fever and signs of neurological manifestations are reported in third week^12^. Another study in bonnet macaques demonstrated, virus-specific gastrointestinal and lymphoid lesions and viral antigens in these same organs by immunohistochemistry in experimentally infected animals^11^. The above studies confirmed the suitability of bonnet macaque as a model to study viscerotropic KFD seen in humans.

Detailed information about multiple aspects of KFD progression with regard to persistence of viremia, time point of first detection, further persistence and titres of anti-KFD IgM and IgG antibodies, viral kinetics and lesions induced in different organs, duration of virus shedding in different secretions and body fluids, biochemical and hematological changes during infection is not available so far. Study of dynamics of various above mentioned parameters, upon inoculation with high and low dose of virus in bonnet macaques was undertaken with the aim to recapitulate the human disease, as bonnet macaques are known to be the only suitable model for KFD studies.

## Results

### Experimental design

**Figure 1:**
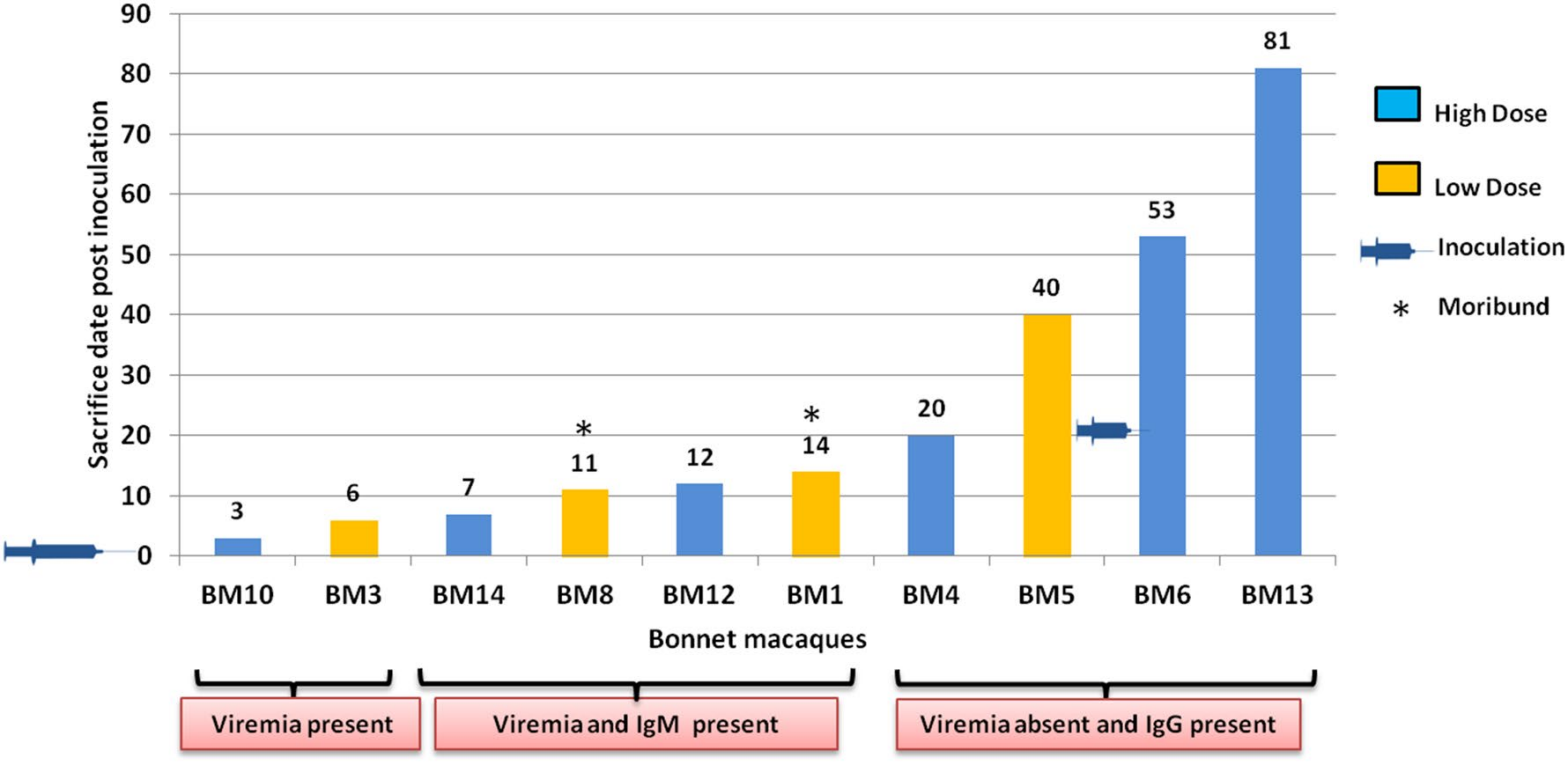
Bonnet macaque sacrifice time points. Each bar (yellow: low dose, blue: high dose) represents the days on which monkeys were sacrificed post KFDV inoculation. Monkeys which became moribund are highlighted with an asterisk. All the monkeys were inoculated with KFDV on day 0 and BM-6 was re-inoculated on day 21.

The experiment was performed for duration of 3 months (March to May, 2018). Bonnet macaques (BM) were randomly assigned into three groups: High dose (Monkey nos: BM4, BM6, BM10, BM12, BM13, BM14), low dose (BM1, BM3, BM5, BM8) and control (BM7). The high dose group was inoculated with 10^5.57^ TCID50 of KFDV, low dose group with 10^3.57^ TCID50 and control with uninfected BHK-21 cell supernatant of the same passage by subcutaneous (s/c) route (1 ml) below the nape of the neck under sedation. Animals were observed twice daily for any clinical signs. Rectal temperature was monitored daily, and body weight was measured every third day post infection (PID). One monkey from each group was sacrificed during (1) viremia, (2) viremia along with IgM response and (3) after the end of viremia along with IgG response **(**Fig. 1**)**. Two macaques, which reached the set humane end points, were sacrificed immediately during the experiment. One macaque was sacrificed on 20th PID, to understand the biphasic nature/neuroinvasion of KFDV and one macaque (BM6) was re-inoculated with 10^5.57^ TCID50 dose on 21st PID. Three macaques (BM-5, BM-6 and BM-13) were kept for longevity study and were sacrificed on 40th, 53rd and 81st PID respectively.

### Clinical findings

In the low dose group, two (BM-5 and BM-8) monkeys developed fever. BM-5 showed the rise in temperature (102 °F–104 °F) from 5th to 9th PID, which dropped to normal (< 102 °F) by 10th PID. Fever***, ***ocular discharge, and watery diarrhoea were observed in BM-8 from 5th PID. The animal became anorexic and exhibited blood tinged diarrhoea from 7th PID. The body condition deteriorated with weight loss of around 13% and was sacrificed on 11th PID. Another monkey from low dose group (BM-1) became anorexic and showed a weight loss of around 8%. On 14th PID, subnormal rectal temperature of 97 °F was noted in BM-1 and was sacrificed.

In the high dose group, BM-14 and BM-13 showed a slight increase in temperature (102 °F) on 4th PID. Signs of dehydration (reduced skin elasticity), reduced activity and anorexia were observed in BM-14. BM-6 showed an increase in temperature up to 103 °F on 11th PID. The other five monkeys (BM4, BM10, BM12, BM3 and BM7) did not show any clinical signs or weight loss throughout the study period.

### Haematology and serum biochemistry

**Figure 2:**
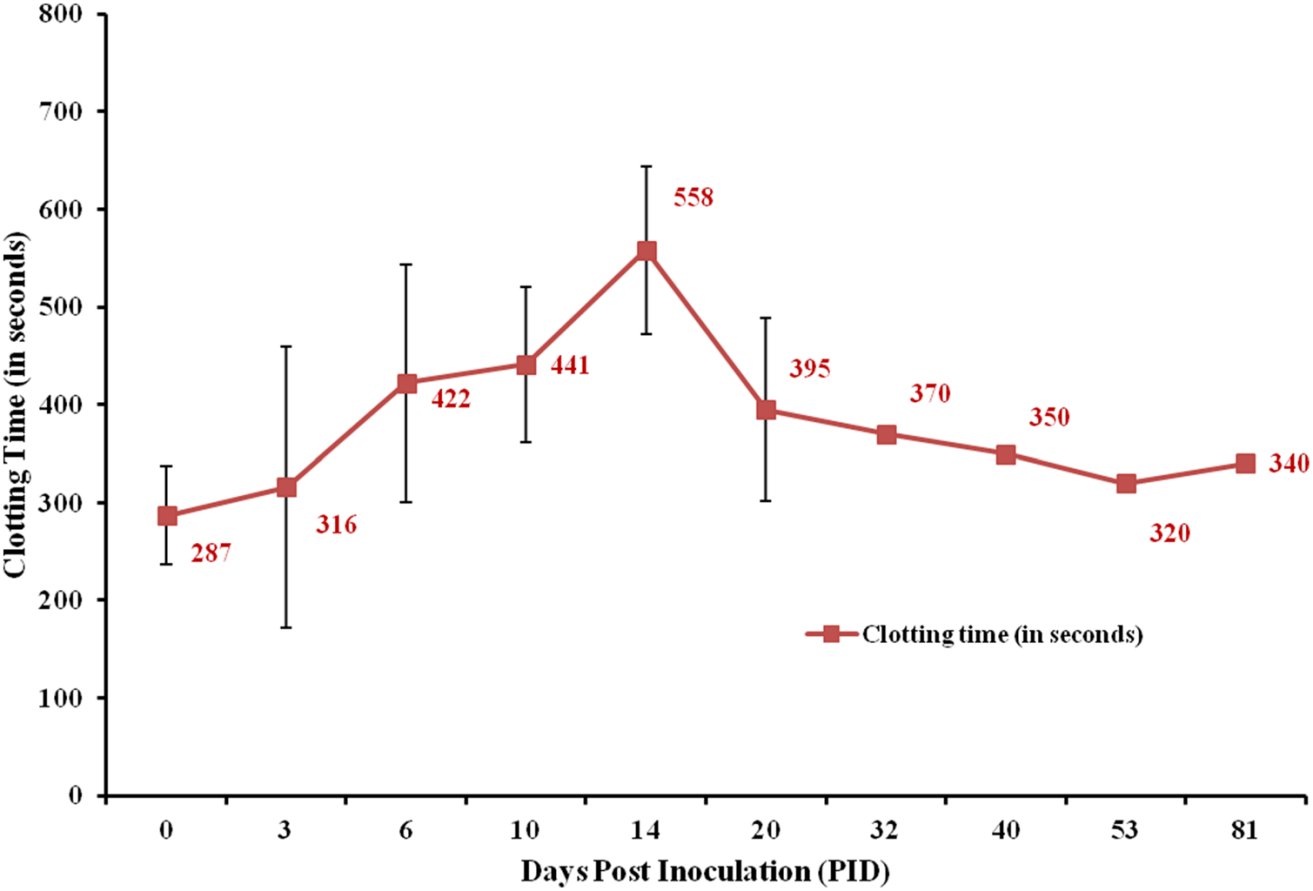
Clotting time. Comparison of average clotting time values at different days post KFDV inoculation. The group sizes at day 0, 3, 6, 10, 14, 20 days post inoculation were 10, 9, 9, 6, 5 and 4 respectively and thereafter 1 animal each.

The blood samples were collected daily from the saphenous vein during the first two weeks post inoculation, which was followed by twice a week collection for the next week and once in a week later till they were sacrificed. From the re-inoculated macaque (BM6), minimum quantity of blood was collected on alternate days from 21st to 32nd PID (10 days post re-inoculation) for testing viremia and antibody response, after which blood was collected once every week till sacrifice. The average clotting time increased progressively from an average of 286.91 s at day 0 to 558 s on 14th PID, after which it gradually reduced to 340 s on 81st PID (Fig. 2). There was no significant change in prothrombin time (PT) and activated partial thromboplastin time (aPTT). No change in clotting time was noted for BM7 (control).

There was no statistically significant variation in any biochemical parameters when data from macaques inoculated with low and high dose; and from moribund and recovered macaques were compared. Therefore, a comparison was made between various time-points. Time points analysed were broadly distributed between day 0, day 3, day 6, day 10, day 14 and 21. The analysis was not performed after 21st PID, as the number of samples collected after that time point were limited (Table 1). There was statistical significant decrease in haemoglobin between day 0 and day 3 (p = 0.0050), and between day 0 and 6 (p = 0.0076). Other factors which showed significant decrease between day 0 and day 3, and day 3 and day 6 were total leukocyte count (p = 0.0051, 0.0077), platelets (p = 0.0069, 0.0108), RBC count (p = 0.0051, 0.0077), PCV (p = 0.0069, 0.0077), neutrophils (p = 0.0151), ALT (p = 0.0128), AP (p = 0.0109), total protein (p = 0.0069, 0.0077), albumin (p = 0.0051, 0.0077), and globulin (p = 0.0069, 0.0209). A significant increase was seen in percent monocytes (p = 0.0322, 0.0276), total bilirubin (p = 0.0438), conjugated bilirubin (p = 0.0236) and AST (p = 0.0218) values between baseline to beginning of viremia, and between the beginning of viremia to the peak of viremia. No significant change was observed between any other time points and other parameters.

**Table 1:** Haematology and serum biochemistry parameters analysed at day 0, 3, 6 and 10 post inoculation.

	Parameter	Mean ± SD				Significance	
		Day 0 (n = 10)	Day 3 (n = 10)	Day 6 (n = 9)	Day 10 (n = 7)	*p* value Day 0 vs Day 3	*p* value Day 3 vs Day 6
1	Hemoglobin, (g/dL)	13.3 ± 1.1	12.2 ± 1.3	11.1 ± 1.1	10.5 ± 1.1	0.0050	0.0076
2	Total leucocytes (/µL)	12,650 ± 3,961	8,120 ± 3,265	5,344 ± 1,165	11,671 ± 4,028	0.0005	0.0077
3	Platelets (/µL)	421,700 ± 85,268	323,000 ± 116,264	279,111 ± 78,731	309,666 ± 161,877	0.0069	0.0108
4	RBC (× 10^6^/µL)	6.6 ± 0.5	6.0 ± 0.7	5.5 ± 0.6	5.1 ± 0.6	0.0051	0.0077
5	PCV (%)	42.8 ± 3.0	39.5 ± 3.7	35.9 ± 3.2	33.7 ± 3.3	0.0069	0.0077
6	MCV (fL)	65.3 ± 5.7	65.8 ± 5.3	65.2 ± 6.0	65.8 ± 4.5	NS	NS
7	MCH (pgms)	20.3 ± 2.1	20.4 ± 2.1	20.2 ± 2.2	20.6 ± 1.9	NS	NS
8	MCHC (g/dL)	31.1 ± 0.8	30.9 ± 1.0	30.9 ± 0.8	31.3 ± 0.9	NS	NS
9	RDW (%)	15.1 ± 1.7	15.3 ± 2.3	14.7 ± 2.2	14.7 ± 1.8	NS	NS
10	Neutrophils (%)	57.6 ± 8.9	62.0 ± 12.8	49.0 ± 9.9	47.9 ± 17.2	0.0151	NS
11	Eosinophils (%)	1.1 ± 1.0	1.3 ± 1.2	0.9 ± 0.8	1.4 ± 1.1	NS	NS
12	Basophils (%)	0.1 ± 0.3	0.2 ± 0.4	0.3 ± 0.5	0.0 ± 0.0	NS	NS
13	Lymphocytes (%)	38.3 ± 8.6	30.4 ± 13.1	42.3 ± 9.2	42.1 ± 14.8	NS	NS
14	Monocytes (%)	2.9 ± 1.0	6.1 ± 3.4	7.4 ± 3.5	8.6 ± 2.2	0.0322	0.0276
15	Bilirubin NS total (mg/dL)	0.1 ± 0.1	0.2 ± 0.1	0.2 ± 0.1	0.2 ± 0.1	0.0438	NS
16	Bilirubin NS conjugated (mg/dL)	0.03 ± 0.02	0.06 ± 0.02	0.05 ± 0.03	0.05 ± 0.01	0.0236	NS
17	Bilirubin NS unconjugated(mg/dL)	0.07 ± 0.0	0.11 ± 0.1	0.12 ± 0.1	0.12 ± 0.1	NS	NS
18	SGOT (AST), (U/L)	28.5 ± 9.0	45.4 ± 18.5↑	49.7 ± 15.9	229.1 ± 398.6	0.0218	NS
19	SGPT (ALT), (U/Lt)	40.8 ± 27.3	42.6 ± 20.4	27.3 ± 9.2	62.0 ± 59.5	0.0128	NS
20	ALP (U/L)	447.1 ± 202.4	453.6 ± 183.5	353.2 ± 147.0	394.0 ± 131.8	0.0109	NS
21	Protein (total) (g/dL)	8.4 ± 0.5	7.5 ± 0.5	5.3 ± 1.8	6.8 ± 0.7	0.0069	0.0077
22	Albumin (g/dL)	3.6 ± 0.2	3.2 ± 0.2	2.6 ± 0.5	2.5 ± 0.3	0.0051	0.0077
23	Globulin (g/dL)	4.9 ± 0.4	4.3 ± 0.3	2.7 ± 1.5	4.4 ± 0.6	0.0069	0.0209
24	Urea (mg/dL)	25.8 ± 4.3	26.0 ± 6.7	25.4 ± 9.8	87.1 ± 161.6	NS	NS

Paired T-tests was used to compare data between various time-points and the *p v*alue of parameters which showed significant difference.

*NS* non significant.

### Presence of anti-KFDV IgM and IgG antibodies

**Figure 3:**
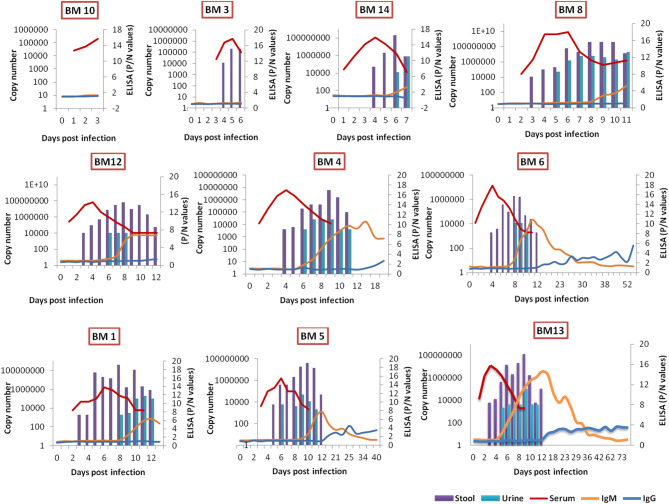
Graphs represent the copy number of KFDV RNA in serum (red line), stool (violet bar) and urine (green bar) (primary y axis) and P/N values of anti-KFDV IgM (orange line) and IgG (blue line) ELISA (secondary y axis) in each macaque at days post KFDV inoculation.

In macaques inoculated with high dose, anti-KFDV IgM and IgG antibodies could be detected from 6th to 42nd PID (peak: 11th–12th PID, OD: 1.147, P/N: 14.6) and 14th PID onwards (peak: 45th PID, OD: 0.632, P/N: 4.22), respectively. In macaques inoculated with a low dose, anti-KFDV IgM and IgG antibodies could be detected from 9th to 34th PID (peak PID: 12, OD: 0.878, P/N: 6.381) and IgG from 18th PID onwards (At PID 40, OD: 0.555, P/N: 3.5) (Fig. 3). The longevity of anti-KFDV IgG could not be estimated beyond day 81 due to experimental limitations. BM7 (control) was negative for anti-KFDV antibodies.

### KFDV RNA in serum, other body fluids, and organs

Viremia could be detected from 1st to 13th PID in high dose group, with a peak on 3rd to 4th PID (Fig. 3). The viral RNA detection in rectal swabs, stool and urine ranged from 3rd to 13th PID, 3rd to 12th PID and 7th to 12th PID, respectively (see Supplementary Table S1). KFDV RNA could be detected in conjunctival swabs and oro-pharyngeal swabs on 10th PID in all KFDV inoculated monkeys. Virus shedding was not observed after re-inoculation in BM-6. In the low dose group, viremia was observed from 3rd to10th PID, and the peak was observed at 6th PID. Rectal swabs and urine were found positive for KFDV RNA from 5th to 12th PID and 6th to11th PID respectively.

The organs that showed the presence of virus from 3rd PID onwards were liver, spleen, ovary and skin. The skin was positive for viral RNA from 3rd PID to 20th PID with increasing viral RNA copy numbers. The lymph nodes were positive with consistently high viral RNA from 3rd to 81st PID followed by stomach, small intestine and large intestine from 3rd PID to 53rd PID. Presence of the virus in the brain was observed only in moribund macaques (BM8, BM1). BM14, sacrificed on 7th PID showed the presence of KFDV RNA in cerebrospinal fluid (CSF) but not in the brain (Table 2).

**Table 2:** Viral load in serum, other body fluids, and organs.

S. no	Organ	Viral RNA copy number									
		BM10 (high, sacrifice: PID3)	BM3 (low, sacrifice: PID6)	BM14 (high, sacrifice: PID7) (dehydrated and lethargic)	BM8 (low, sacrifice: PID 11) (Moribund)	BM12 (high, sacrifice: PID12)	BM1 (low, sacrifice: PID14) (Moribund)	BM4 (high, sacrifice: PID 20)	BM5 (low, sacrifice: PID 40)	BM6 (high, sacrifice: PID 53)	BM13 (high, sacrifice: PID 81)
1	CSF	Negative	Negative	1 × 10^4^	6 × 10^4^	Negative	3 × 10^4^	Not collected	Negative	Negative	Negative
2	Cerebellum	Negative	Negative	Negative	1 × 10^9^	Negative	2.5 × 10^5^	Negative	Negative	Negative	Negative
3	Cerebrum	Negative	Negative	Negative	3 × 10^7^	Negative	Negative	Negative	Negative	Negative	Negative
4	Olfactory bulb	Negative	Negative	Negative	1 × 10^4^	Negative	Negative	Negative	Negative	Negative	Negative
5	Hippocampus	Negative	Negative	Negative	4 × 10^6^	Negative	Negative	Negative	Negative	Negative	Negative
6	Medulla oblongata	Negative	Negative	Negative	Not collected	Negative	6 × 10^4^	Negative	Negative	Negative	Negative
8	Pancreas	Negative	1 × 10^4^	3 × 10^7^	8 × 10^6^	Negative	6 × 10^8^	2.5 × 10^5^	1.2 × 10^5^	6 × 10^4^	Negative
9	Liver	3 × 10^4^	2.5 × 10^5^	8 × 10^6^	4 × 10^6^	Negative	3 × 10^4^	2.5 × 10^5^	Negative	Negative	Negative
10	Spleen	6 × 10^4^	2.5 × 10^4^	3 × 10^7^	4 × 10^10^	3 × 10^7^	1 × 10^9^	3 × 10^7^	2.5 × 10^5^	2.5 × 10^5^	Negative
11	Kidney	Negative	2.5 × 10^4^	3 × 10^4^	3 × 10^7^	Negative	1.2 × 10^5^	6 × 10^4^	Negative	Negative	Negative
12	Heart	Negative	2.5 × 10^4^	3 × 10^4^	3 × 10^7^	Negative	1.5 × 10^4^	Negative	Negative	Negative	Negative
13	Lungs	Negative	2 × 10^4^	1.2 × 10^5^	3 × 10^8^	Negative	1 × 10^6^	1.2 × 10^5^	Negative	Negative	Negative
14	Gall bladder	Negative	2 × 10^4^	2 × 10^6^	6 × 10^4^	Negative	6 × 10^4^	Negative	Negative	Negative	Negative
15	Bile	Negative	Negative	Negative	1 × 10^6^	Negative	Negative	Negative	Negative	Negative	Negative
16	Stomach	5 × 10^5^	1 × 10^6^	5 × 10^9^	1 × 10^9^	7 × 10^7^	3 × 10^7^	6 × 10^4^	2.5 × 10^4^	1 × 10^4^	Negative
17	Small intestine	2.5 × 10^5^	4 × 10^6^	3 × 10^7^	1 × 10^9^	2.5 × 10^5^	6 × 10^4^	2 × 10^9^	3 × 10^4^	1 × 10^4^	Negative
18	Large intestine	5 × 10^5^	5 × 10^8^	6 × 10^8^	6 × 10^8^	1 × 10^7^	3 × 10^8^	5 × 10^5^	2.5 × 10^4^	6 × 10^4^	Negative
19	Mesentric lymph node	5 × 10^5^	5 × 10^9^	4 × 10^10^	8 × 10^6^	1 × 10^8^	5 × 10^9^	Not collected	1 × 10^7^	3 × 10^7^	4 × 10^6^
20	Ovary	3 × 10^4^	1.2 × 10^5^	3 × 10^4^	8 × 10^6^	Negative	2.5 × 10^5^	6 × 10^4^	Negative	Negative	Negative
21	Back skin	6 × 10^4^	1.5 × 10^4^	1 × 10^6^	250,000	Negative	1.2 × 10^5^	6 × 10^8^	Negative	Negative	Negative
22	Urinary bladder	Negative	2.5 × 10^4^	2 × 10^6^	2 × 10^6^	Negative	6 × 10^4^	Negative	Negative	Negative	Negative
23	Urine	Negative	Not collected	8 × 10^6^	Negative	Negative	Negative	Negative	Negative	Negative	Negative
24	Serum	10^6^	5 × 10^5^	6 × 10^4^	8 × 10^6^	Negative	Negative	Negative	Negative	Negative	Negative
25	Throat swab	Negative	1.5 × 10^4^	3 × 10^4^	1.5 × 10^4^	Negative	1 × 10^4^	Negative	Negative	Negative	Negative
26	Conjunctival swab	Negative	Negative	1.5 × 10^4^	1 × 10^7^	Negative	1 × 10^4^	Negative	Negative	Negative	Negative
27	Rectal swab/stool	Negative	1 × 10^6^	4 × 10^6^	1 × 10^8^	3 × 10^4^	1 × 10^6^	1.5 × 10^4^	Negative	Negative	Negative

### Virus isolation from skin

The infant mice litters inoculated with monkey skin suspensions of BM10 (sacrificed on 3rd PID) and BM14 (sacrificed on 7th PID) showed sickness. Brain suspensions of sick mice were found positive for KFDV RNA with 1.3 × 10^12^ (BM10) and 2.7 × 10^12^ (BM14) copy numbers.

### Histopathology

**Figure 4:**
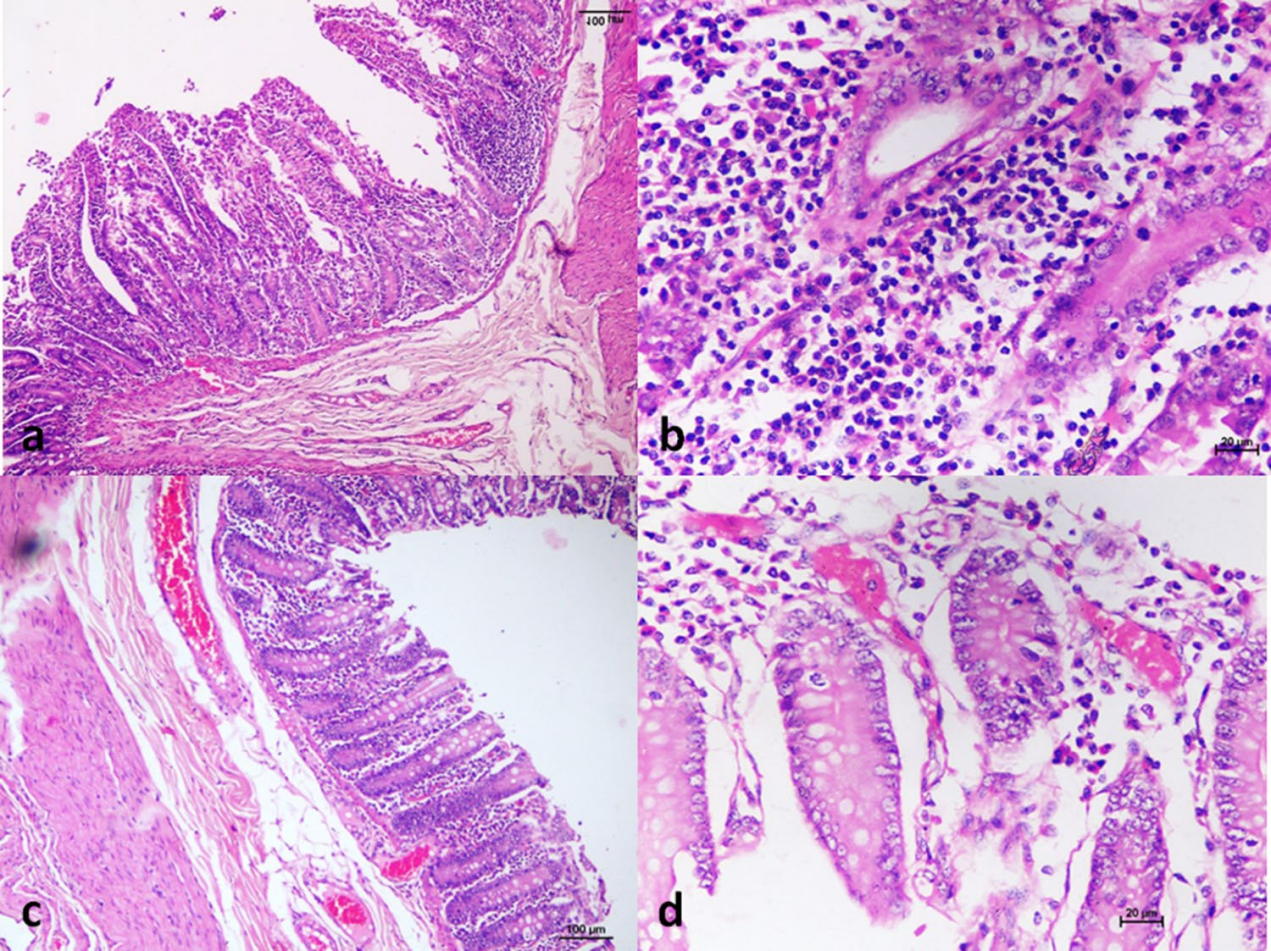
Histopathological observations. **(a)** Mucosal ulceration and focal mononuclear infiltration in small intestine, H&E, × 100, **(b)** mononuclear cell infiltration in the small intestine, H&E × 200, **(c)** sub-mucosal congestion in the large intestine, H&E × 100, **(d)** submucosal congestion in the large intestine, H&E, × 200.

The histopathological changes observed were minimal irrespective of the dose of inoculation, and majority of the changes were observed in the gastrointestinal tract (GIT). Mucosal ulceration and focal/mild mononuclear cell infiltration were observed in stomach (BM-3, BM-4, BM-6, BM-8, BM-10, BM-14), small intestine (Fig. 4a, b) (BM-3, BM-4, BM-10, BM-14) and large intestine (BM-1, BM-3, BM-6, BM-10, BM-13, BM-14, BM-5). Mild sub-mucosal congestion was also observed in the large intestine (Fig. 4c, d) in BM-1, BM-4, and BM-8. Kidneys showed inflammatory cell infiltration in the cortex and medulla in BM-1, BM-5, BM-6, BM-8 and BM-14. Other organs did not show any marked histopathological changes except few focal degenerative foci in liver in BM-8 and BM-14.

### Immunohistochemistry

**Figure 5:**
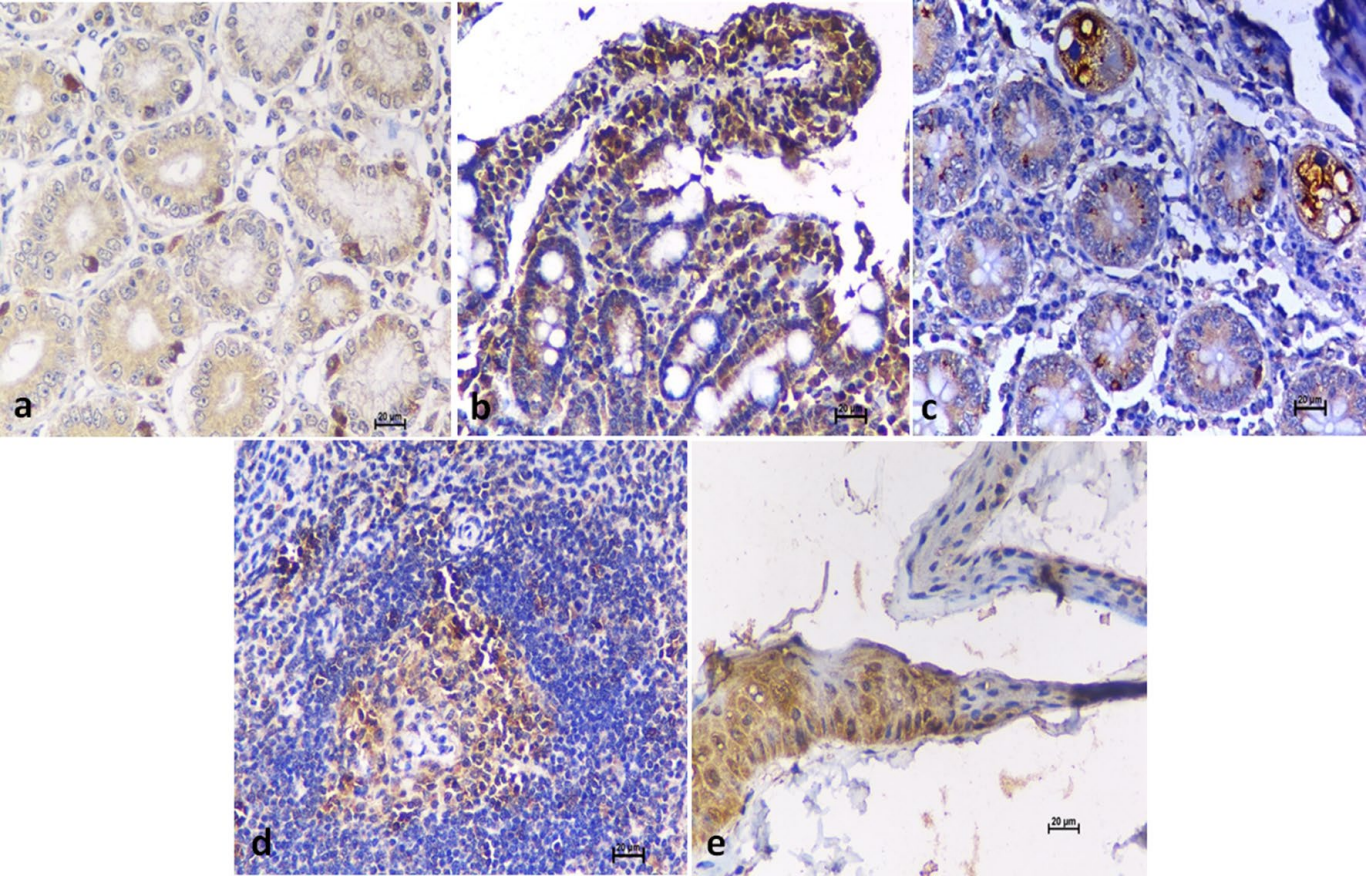
Immunohistochemistry. **(a)** Stomach: cytoplasmic immunostaining of glandular cells, DAB × 200, **(b)** Small intestine: immunostaining of intestinal mucosa, DAB × 200. **(c)** Large intestine: immunostaining of epithelium, DAB × 200, **(d)** Spleen: immunostaining of splenic white pulp, DAB × 200, **(e)** Skin: immunostaining of epidermis layer, DAB × 200.

In case of high dose virus inoculated monkeys, positivity was observed in monkeys sacrificed on 3rd, 7th, 12th and 53rd PID in stomach (Fig. 5a) and small intestine (Fig. 5b). Large intestine showed immunostaining in BM-3, BM-6, BM-8 and BM-14 (Fig. 5c). In case of monkeys inoculated with low dose and sacrificed on 6th, 11th and 14th PID, cytoplasmic immunostaining was observed in stomach, small intestine and spleen (Fig. 5d). Skin positivity was observed in monkeys sacrificed on 3rd, 6th, 7th, 11th, 14th and 53rd PID (Fig. 5e).

### Cytokine analysis

**Figure 6:**
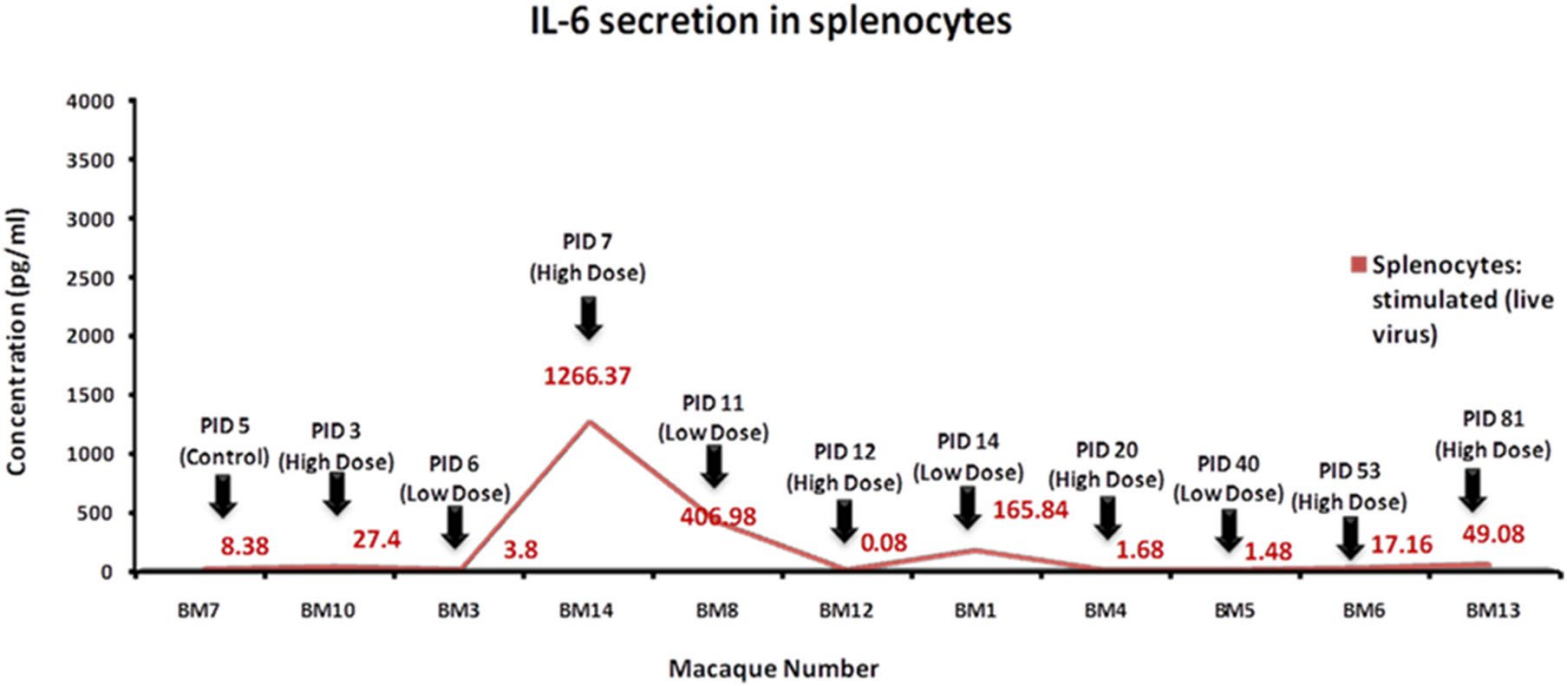
Cytokine analysis. Profile of IL-6 secretion of KFDV stimulated splenocytes in different macaques at sacrifice.

Out of the six cytokines analyzed in stimulated splenocytes, only IL-6 could be detected. A clear pattern of IL-6 secretion was observed from 7th to 14th PID in splenocytes of BM14, BM8 and BM1. The cytokine secretion was found minimal in BM12. Non-stimulated splenocytes did not show any cytokine secretion (Fig. 6).

### Taqman low density array

Out of the 92 genes of the immune panel studied, 15 genes showed marginal fold change (see Supplementary Fig. S1). The fold change did not show any specific trend across various points of sacrifice. Pro-inflammatory cytokines showing up regulation were IL15 (BM12, BM4) and Colony Stimulating Factor 3 (CSF3) (BM10, BM3, BM14, BM8, BM4, BM5).T helper 1 (Th1) specific genes that showed fold increase were IFNγ (BM5), IL18 (BM1, BM4), IL2 (BM1, BM4), IL12A (BM10, BM1). IL2 was found marginally down-regulated in BM8. Th2 specific genes which showed up regulation were Cytotoxic T Lymphocytic Associated Protein-4 (CTLA4) (BM10, BM5, BM6), Human Leukocyte Antigen – DR isotype A (HLA-DRA) (BM14, BM4) and IL13 (BM8, BM12, and BM1) were found to be down regulated. Other genes that showed marginal fold change were Intercellular Adhesion molecule 1 (ICAM1), Selectin P (SELP), Selectin E (SELE),and Fas ligand (FASLG).

Overall the pro-inflammatory genes, namely CSF3, chemokine (C-X-C motif) ligand 10(CXCL10), and SELE, were found consistently marginally elevated in all monkeys except BM12. Th1 genes, namely, IL2, IL12A, IL18, IFN-gamma, and IL15 were found elevated from 14^th^PID to 40^th^ PID. IL13 was marginally down regulated from 11^th^ PID to 14^th^ PID. Supportive Th2 genes, namely CTLA4 and HLA-DRA were marginally elevated, though not consistently. Other genes showing increased fold change were SELP, cluster of differentiation (CD) 8A, ICAM1. Gene down-regulated at 11^th^PID in the moribund macaque was FAS-LG. Overall; the immune response pattern was balanced with marginal skewing towards pro-inflammatory and Th1 response.

## Discussion

KFD is an important emerging infection in India, as evident by the spread of the disease to newer geographic areas, particularly during the last decade^2^. Experimental infection studies in animals have been conducted in the past using prototype old isolate maintained by serial passaging in suckling mouse brain^11,12,14–16^. Arboviruses are maintained in nature via alternate cycles of replication in host and vector. Serial passaging in single host may lead to adaptation owing to increase in mutation rates, a phenomenon commonly observed in arboviruses^18^. Using such a virus in animal models may not represent true disease profile in natural settings^16^. Besides that, change in genetic makeup of contemporary virus over a period of time may lead to differential disease outcome compared to the isolates obtained during 1960s^19^. A recent study on the phylogeography of the KFDV also has shown only 0.86 percent amino acid divergence between KFD recent and older isolates^20^. We have studied experimental infection in bonnet macaques with contemporary, minimally passaged strain so as to mimic the scenario observed in nature. Unlike 100% mortality observed in previous macaque studies^11,12^, we observed severe disease in 2/10 macaques. However, the similar low passage strain of KFDV used in an earlier study produced neuropathology in CD1 mice^21^. The dose of infection in the present study has also not shown any effect on mortality. Level and duration of viremia in macaques was found to be comparable with the previous studies^11,12^. In spite of considerable and prolonged viremia, disease in 2/10 macaques indicates that apart from virus strain, host cofactors could play important role in disease outcome. Indeed, the host factors leading to the alteration in immune response are known to affect disease outcome in KFD patients^22^.

Our study revealed that infected macaques can be asymptomatic and shed the virus in stool, urine, oral and conjunctival secretions during the viremic phase. The transmission of the virus in nature from macaque to macaque via bites, open wounds, or direct contact with contaminated fomites could be possible. Moreover, this could act as source of virus to small mammals via contaminated fomites. A decade long study conducted on monkey mortality in KFD endemic area revealed that, out of 1,046 deaths, 860 were *P. entellus* and only 186 were *M. radiata* with virus isolation percentage of 50% and 18.05% in necropsied animals respectively^17^. The findings suggest that bonnet macaques are either not highly susceptible to virus or many macaques could be sub clinically infected. We observed that all infected macaques developed considerable viremia and robust humoral immune response indicating that the latter may be true. In our re-infection study, viremia or virus shedding was not observed, indicating that protective humoral immune response was generated which could counter high dose of virus during re-infection. This is in agreement with the earlier report stating that many macaques in wild survive the unapparent infections due to presence of neutralising antibodies^23^. In agreement with other macaque studies, fever was not consistently observed in infected animals. Few animals (3/10) exhibited rise in body temperature transiently. Only one animal in which severe disease was observed showed consistent fever. Haemorrhage is not consistent feature of KFD. GIT haemorrhages were observed in patients only with severe disease^22^. Indeed we could observe frank GIT haemorrhages in macaque with severe disease. None of the animals developed biphasic fever or neurological sequelae although viral RNA could be detected in brains of two macaques with severe disease.

KFD viral RNA in blood could be detected from 1st–13th PID in macaques inoculated with high dose with peak at 4th PID. In macaques inoculated with low dose, detection and peak was delayed by 2 days. Importantly, the peak RNA levels in a macaque with fatal disease was significantly higher than other animals indicating that level of viremia could be a marker of severe disease. Overall pattern of viremia was very similar to that observed in patients and other macaque studies^11,12,24^. Of note, the detection of viral RNA in faeces/rectal swab coincided with the peak of viremia and continued until the end of viremia. Detection in urine samples was invariably delayed by at least two days. This probably reflects on the primary replication site for the virus. GIT has been shown to be affected in rodent and macaque models^11,14^. In the macaque sacrificed on 3^rd^ PID, viral RNA could be detected in GIT but not in kidneys or urinary bladder. The experimental studies on faecal detection of Zika virus RNA in neotropical primates is considered a significant advancement for surveillance of Zika in wild reservoir monkeys^25^. The faecal detection of KFDV RNA could turn out to be a cost effective, non invasive and reliable method to monitor wild monkeys during sentinel surveillance. Viral RNA was also detected in conjunctival swabs and pharyngeal swabs collected on 10th PID. Ocular manifestation in KFD in patients is very common^26^. Virus shedding in different secretions / excretions has not been studied in patients so far. Such a study would be useful in identifying non invasive specimens to be used in diagnosis / follow up with patients ^2^.

Kinetics of viral RNA in different organs of macaques was studied throughout the course of disease. Viral RNA could be detected in liver, spleen, GIT, ovary and skin of the macaque sacrificed on 3rd PID and dissemination to other visceral organs was observed in the macaque sacrificed on 6th PID. The findings are in agreement with earlier macaque study in which GIT, organs of lymphoreticular system and liver were found as primary replication sites for the virus^11^. Earlier rodent model study also reported spleen and GI tract beside brain as the replication site for the virus^14^. Viral RNA in liver, heart, lungs, bladder, ovary, skin could be detected in macaques sacrificed from 6th PID until 20th PID. Thereafter it could be detected in liver, spleen, GIT and lymph nodes in the macaques sacrificed on 40th and 53rd PID and exclusively in lymph node, in the macaque sacrificed on 81^st^ PID. Further studies are needed to ascertain replication competency of the persistant viral RNA in GIT and lymph nodes. Persistence of replication competent virus in lymph nodes have been observed in macaques experimentally infected with Zika virus^27^. Only two monkeys which reached the moribund state showed positivity in brain samples suggesting KFDV may affect central nervous system (CNS) at a later stage of infection (11th to 14th PID). Our observations are in unison with earlier studies which reported encephalitis not evident before 12th PID^13,28^.

Humoral immune response against KFDV in animal model has not been studied so far owing to the acute nature of disease in rodents and macaque models^11,14,16^. We could study the antibody kinetics over a period of 80 PID. Until 40 PID, at least three animals could be sampled per time point, after that there were only two animals on day 53 and one on day 80. Positive titres of IgM ( P/N > 2) could be detected from the end of first week post infection until 5 weeks post infection, with peak titres coinciding with end of viremia (12 PID). IgG could be detected from 3rd week onwards with peak titres by 6 weeks post infection. Positive titres of IgG could be detected throughout the observation period. The peak IgM was observed on 12th PID (end of viremia) suggesting that IgM efficiently neutralised free virus even before the detectable IgG appeared in the blood. Considerable overlap of at least 6 days was observed in viremia and IgM. Webb and Chatterjea, 1962 has shown antibodies on 65th PID in *M. radiata* inoculated with KFDV but other time points data was unavailable till date^28^.

Alterations in hematological parameters were observed in percent monocytes, thrombocytes, total leukocytes and erythrocytes. We observed increase in monocytes with concomitant decrease in thrombocytes and total leukocytes during viremic phase. Increase in absolute number of monocytes within first 5 days after infection was observed in macaques infected with Zika virus^29^. Transient lymphopenia as observed in our study is commonly observed in viral infections and has been shown to be associated with an early Type I interferon response^26^. Our finding of increased monocytes with concomitant decrease in erythrocytes is in agreement with elevated myeloid/erythroid ratios observed in KFDV infected macaques by an earlier study^11^. Marked thrombocytopenia and anaemia, possibly due to erythrophagocytosis were observed earlier in KFD^13^. A significant decrease was observed in the total protein, globulin and albumin levels points to altered liver function which is supported by an increase in AST and bilirubin levels. But no histopathological lesions were observed in liver. Anorexia or lesions in the GIT (although minimal) contributed by the viral replication might have resulted in protein malabsorption which could also result in decreased serum proteins. High AST level in KFD affected monkeys was earlier demonstrated^11^.

The IgM antibody rise corresponded to the IL-6 secretion in splenocytes. IL-6 is a multifunctional cytokine with a wide range of functions important in inflammation and immune response^30^. However, the relation cannot be confirmed due to the limited sample size. In contrast, the IL-6 secretion was minimal in BM12 sacrificed on 12th PID. BM12 also had not shown the presence of KFDV RNA in any organs except spleen, stomach and intestines. Gene expression analysis showed only a marginal fold change in few pro-inflammatory and Th1 genes in comparison to the control monkey. There was a limitation of sample size at different time points.

We have attempted to recapitulate KFD in the macaque model using high and low doses of minimum laboratory passaged, the contemporary strain of virus by closer to natural route (s/c) of inoculation mimicking the tick bite. Majority of the macaques showed minimal clinical signs and the mortality was comparable to humans. Neuro-invasion was observed only in the case of moribund animals without the evidence of encephalitis. Infected macaques were found to shed the virus in stool, urine, oral and conjunctival secretions during viremic phase. Importantly, virus could be isolated from skin during period of viremia. Viral load study in different organs suggested that lymphoid organs and GIT could be the major replication/persistence sites. Viremia, virus shedding in different secretions/ excretions, antibody response, viral RNA load observed in experimentally infected macaques could be used as markers in evaluation of future NHP models.

## Materials and methods

### Ethics statement

The study (Project no. MCL-1503) was recommended by the Institutional Animal Ethics Committee, Indian Council of Medical Research (ICMR)-National Institute of Virology (NIV), Pune and was approved by the Committee for the purpose of Control and Supervision of Experiments on Animals (CPCSEA), letter F No. 25/28/2016-CPCSEA dated 02.12.16. The permission from forest authorities for trapping of required number of bonnet macaques from the wild was also obtained. The research was conducted in compliance with the guidelines laid down by CPCSEA, Government of India.

### Virus

KFDV (NIV-12839) isolated from acute phase human serum sample from Thirthahalli, Karnataka propagated in Baby hamster kidney (BHK) -21 cells (Passage number 6) having a tissue culture infective dose 50 (TCID50) titer of 10^5.57^/ml was used for the study^21^**.** 6–8 weeks old, female, BALB/c mice procured from Animal House facility of ICMR-NIV, Pune was used to assess the virus lethal dose 50 (LD50). The animals were housed in individually ventilated cages in the Animal Biosafety level-3 (ABSL-3) facility. Ten-fold serial dilutions of virus stock in minimum essential medium (MEM) (Life Technologies Corporation, USA) was prepared. Six mice were used per dilution and the mice were inoculated by s/c route at a dose of 100 micro L (µl)/mouse. Mice were monitored for 21 days for mortality, and LD50 value was calculated by Reed and Muench method^31^. The mouse LD50 titre of the KFD virus following subcutaneous inoculation in 6–8 week old BALB/c mice was found to be 10^5.5^/ml.

### Bonnet macaques

Eleven, female, adult bonnet macaques weighing 4 to 5.5 kg were caught from the Pune Forest Division of Maharashtra State, India and were kept under quarantine at the animal house facility at the ICMR-NIV, Pune for two months. During the quarantine, veterinary clinical examination, haematology, serum biochemistry, stool examination for parasites and pathogenic bacteria, chest X-ray, tuberculin tests and anti-KFD monkey IgG ELISA were performed to judge if the monkeys were fit for the experiment. After the successful quarantine, the monkeys were shifted to ABSL-3 facility and kept for an acclimatization period for one week. Animals were housed in individual stainless steel cages, fed twice daily with commercial feed pellets, vegetables, fruits, nuts and were given ad libitum access to drinking water. The humane end point for the experiment was decided based on the available literature^32^, and the monkeys were euthanized by intravenous administration of thiopentone sodium under deep sedation with ketamine hydrochloride^33^. The humane end points were determined based on their general behaviour, skin condition, body weight, excessive nasal/ocular discharge, diarrhea, vomiting, urination, and body temperature.

### Occult blood test

The occult blood test was performed for stool using a commercially available test kit (Hemospot kit, Coral Clinical systems).

### Haematology and serum biochemistry

Coagulation parameters were measured every third PID. Clotting time (CT) was assessed using the capillary method. Blood collected in 4% sodium citrate solution was used for testing PT and aPTT using the commercially available human kit (Tulip Diagnostics (P) Ltd).The samples collected on every third day post inoculation were outsourced to a commercial National Accreditation Board for Testing and Calibration Laboratories accredited private laboratory of Pune. Haemoglobin (Hb), total leucocyte count (TLC), differential leucocyte count (DLC), platelet count, packed cell volume (PCV), mean corpuscular volume (MCV), mean corpuscular haemoglobin (MCH), mean corpuscular haemoglobin concentration (MCHC) and red blood cell count were tested. Bilirubin, aspartate aminotransferase (AST), alanine aminotransferase (ALT), alkaline phosphatase (AP), total protein, albumin, globulin, urea and creatinine were assessed.

### Measurement of anti-KFDV antibody response by in-house ELISA

Indigenous serological tests for detecting anti-KFDV IgM and IgG antibodies from monkey sera were developed. The intra-assay and inter-assay variation were determined. The cut-off value was determined by screening normal monkey sera and the sample was considered positive if the Optical Density (OD) value is > 0.2 and the Positive/Negative (P/N) ratio > 1.5 for both the assays.

#### Anti-KFD monkey IgM detection ELISA

ELISA plates were coated with anti-monkey IgM antibodies (1:100, Sigma SAB 3700778) in carbonate buffer (pH 9.2, 0.025 M) overnight at 4 °C. The wells were blocked with 2% bovine serum albumin (BSA) for 2 h at room temperature. One hundred µl of a diluted sample (1:100 in 1% BSA in 1 × phosphate-buffered saline (PBS) containing 0.1% Tween20) was added to duplicate wells and incubated at 37 °C for one hour. One hundred µl of inactivated KFDV antigen (strain number: 12839) was used as positive antigen; normal BHK-21 cell lysate was used as negative antigen, which were added to the wells and the plates were incubated for one hour at 37 °C. Anti-KFDV biotinylated antibodies (100 µl, 1:2000) were added and incubated for one hour. Streptavidine Horseradish peroxidase (HRP) (100 µl; 1:8,000) was added and incubated for 30 min at 37 °C. Each incubation was followed by 4 washes with wash buffer (1 × PBS containing 0.1% Tween). One hundred µl of 3, 3′,5,5′-Tetramethylbenzidine (TMB) substrate was added and incubated for 10 min. The reaction was stopped by 1 N Sulphuric acid (H_2_SO_4),_ and the absorbance was measured at 450 nm (nm).

#### Anti-KFD monkey IgG detection ELISA

ELISA plates were coated with inactivated KFDV antigen (1:10) in carbonate buffer (pH 9.2, 0.025 M) overnight at 4 °C. One hundred µl of 1:100 diluted serum samples were added and incubated for 1 h at 37 °C. Anti-monkey IgG HRP conjugate (1:4,000, 100 µl) was added and incubated for one hour at 37^0^C. The plate was washed four times with wash buffer, after the completion of each incubation period. One hundred µl of TMB substrate was added and incubated for 10 min. The reaction was stopped by 1 N H_2_SO_4,_ and absorbance was measured at 450 nm.

### Measurement of KFDV RNA load by real-time quantitative reverse transcription PCR (qRT-PCR)

Fresh stool/rectal swabs and urine were collected daily. Conjunctival and oro-pharyngeal swabs were collected under sedation. CSF was collected from all macaques by cisterna magna puncture under deep anaesthesia just before sacrifice. All the visceral organs, brain, and skin were collected during necropsy in RNAlater (Sigma), MEM and 10% neutral buffered formalin for further studies.

Viral RNA load was determined in serum, urine, stool/ rectal swabs, oro-pharyngeal swabs, conjunctival swabs, bile, CSF and in organs like skin, heart, lungs, liver, gall bladder, spleen, kidney, urinary bladder, pancreas, stomach, small intestine, large intestine, lymph nodes, ovary, cerebellum, cerebrum, olfactory bulb, hippocampus and medulla oblongata. A 10% homogenate of uniformly weighed tissues in 1 ml of MEM was used for RNA extraction using Magmax RNA extraction kit (Thermo Fisher Scientific, USA) according to manufacturer’s instructions. KFDV qRT-PCR was performed using published primers and probes^34^.

### Virus isolation from skin

Skin samples (below the nape of neck) were collected during necropsy. Infant CD1 mice (1 day old) were inoculated intra-cerebrally with 20 µl of filtered monkey skin suspension prepared in MEM. Mice were observed daily and reduced mobility, hunched posture, paralysis and laboured breathing were considered humane end-points. Mice brains were harvested in aseptic conditions on observation of sickness on 7^th^ PID. 10% of mouse brain suspension was made in 1.25 molar PBS with 10% bovine albumin and tested for the presence of KFDV RNA by qRT-PCR.

### Histopathology

Representative tissue samples of heart, lungs, liver, spleen, kidneys, stomach, small and large intestine, pancreas, lymph nodes, urinary bladder, brain and skin were immersion-fixed in 10% neutral buffered formalin. Tissues were processed by routine histopathology technique, embedded in paraffin, and sections of 4 µm (μm) thickness were cut for subsequent haematoxylin and eosin staining^35^.

### Immunohistochemistry

Formalin fixed tissue sections taken on adhesive coated slides were re-hydrated, and antigen retrieval was performed in 0.3% hydrogen peroxide solution in methanol. Further, the sections were blocked with blocking buffer for one hr at 37 °C. The sections were exposed to primary antibody (1:400 dilutions of polyclonal sera against KFDV raised in mice) for one hour at 37 °C followed by HRP labelled secondary antibody. Immuno-reactivity was detected using 3, 3′-diaminobenzidine tetrahydrochloride substrate, and hydrogen peroxide. Sections were counterstained with Haematoxylin stain.

### Cytokine analysis by cytometric bead array

Cytokine analysis was performed as described previously^36^. Briefly, one million freshly harvested splenocytes from sacrificed monkeys were stimulated with 10^5^mouse LD50 live KFDV. The stimulated cells were incubated at 37 °C for 72 h, centrifuged and the supernatant containing secreted cytokines was further used. Six cytokines, namely IL -2, IL-4, IL-5, IL-6, TNF and IFN-γ were measured using cytometric bead array non-human primate Th1/Th2 kit (BD Biosciences San Jose, CA, USA).

### Gene expression analysis by Taqman low density array (TLDA)

Taqman based analysis was performed on spleen tissues as previously described^37^. RNA was extracted using the Qiagen total RNA extraction kit (Qiagen, Hilden, Germany) and was quantified using the Qubit RNA HS assay kit (Thermo Fisher Scientific, Carlsbad, California, USA) according to manufacturer’s instructions. The quality and integrity of total RNA were confirmed by checking the presence of 18S (ribosomal) rRNA and 28S rRNA bands on an agarose gel. One microgram total RNA was used for complementary deoxy ribonucleic acid (cDNA) synthesis using High-Capacity cDNA Reverse Transcription Kit (Thermo Fischer Scientific). The quality of cDNA was confirmed using real time PCR set up with eukaryotic 18S rRNA endogenous control (Thermo Fisher Scientific, Carlsbad, California, USA). TLDA plate (Applied Biosystems, UK) of the immune panel with 10 ng of cDNA per reaction was run on the 7,300 Real-time PCR system (Applied Biosystems, UK). Hypoxanthine phosphoribosyl transferase (Hprt)1 gene (housekeeping gene) was used as endogenous control. Relative gene expression values were calculated with the comparative cycle threshold (Ct) method using Applied Biosystems relative quantification (RQ) manager software v1.2.cDNA from the control monkey (BM7) was considered as the calibrator. A fold change greater or less than 2.5 was considered valid in the experiment.

### Statistical analysis

Descriptive statistics were calculated for quantitative variables, mean, and standard deviation. For qualitative variables, percentages were calculated. Paired T-tests was used to compare data between various time-points. Statistical significance was considered at 5% level of significance (*p* value < 0.05).

## Supplementary information

Supplementary file1 (PDF 267 kb)

## Data Availability

The authors confirm that the data supporting the findings of this study are available within the article or its supplementary materials.
