# Supplementary material for: Study of Kyasanur forest disease viremia, antibody kinetics, and virus infection in target organs of Macaca radiata
Source: Sci Rep. 2020 Jul 28;10:12561. doi: 10.1038/s41598-020-67599-x (PMC7387489; doi:10.1038/s41598-020-67599-x)
Supplement: Supplementary file 1 — Supplementary file1 (PDF 267 kb) [file 41598_2020_67599_MOESM1_ESM.pdf]

Study of Kyasanur Forest Disease viremia, antibody kinetics, and virus infection in target organs of *Macaca radiata*

Authors: Dilip R Patil[1¶], Pragya D Yadav[1¶], Anita Shete[1&], Gouri Chaubal[1&], Sreelekshmy Mohandas[1&], Rima R Sahay[1&],Rajlaxmi Jain[1&], Chandrashekhar Mote[2&], Sandeep Kumar[1&], Himanshu Kaushal[1#], Pravin Kore[1#], Savita Patil[1#], Triparna Majumdar[1#], Siddharam Fulari[1#], Annasaheb Suryawanshi[1#],Manoj Kadam[1#], Prachi G Pardeshi[1#], Rajen Lakra[1#], Prasad Sarkale[1#], Devendra T Mourya[1*]

First Author [¶], 2nd author [&], 3rd author [#]

[¶] The authors (Dilip R Patil, Pragya D Yadav) contributed equally to this work.

[&] The authors (Anita Shete, Gouri Chaubal, Sreelekshmy Mohandas, Rima R Sahay, Rajlaxmi Jain, Chandrashekhar Mote, Sandeep Kumar) also contributed equally to this work.

[#] The authors (Himanshu Kaushal, Pravin Kore , Savita Patil, Triparna Majumdar, Siddharam Fulari, Annasaheb Suryawanshi, Manoj Kadam, Prachi G Pardeshi, Rajen Lakra, Prasad Sarkale) also contributed equally to this work.

Supplementary Table S1: Period of detection of KFDV RNA in serum and other body fluids.

Virus Dose	Monkey number	Viremia (PID)			Copy number at peak viremia	Viral RNA detection range (PID)	
		Start	Peak	End		Stool	Urine
High dose ($10^{5.5}$ $TCID_{50}$)	BM10	1	-	Sacrificed on 3rd PID	-	-	-
	BM14	1	4	Sacrificed on 7th PID	$6x10^8$	4-7	6-7
	BM12	1	4	Sacrificed on 12th PID	$7x10^7$	3-12	6-8
	BM4	1	4	11	$7x10^7$	4-11	6-11
	BM13	1	3	11	$7x10^7$	3-12	5-11
	BM6	1	4	11	$1x10^7$	4-12	8-10
Low dose ($10^{3.5}$ $TCID_{50}$)	BM3	3	5	Sacrificed on 6th PID	$8x10^4$	4-6	5
	BM8	2	6	Sacrificed on 11th PID	$4x10^{10}$	3-11	5-11
	BM1	3	6	11	$8x10^6$	3-12	8-11
	BM5	3	6	10	$8x10^6$	5-12	6-11

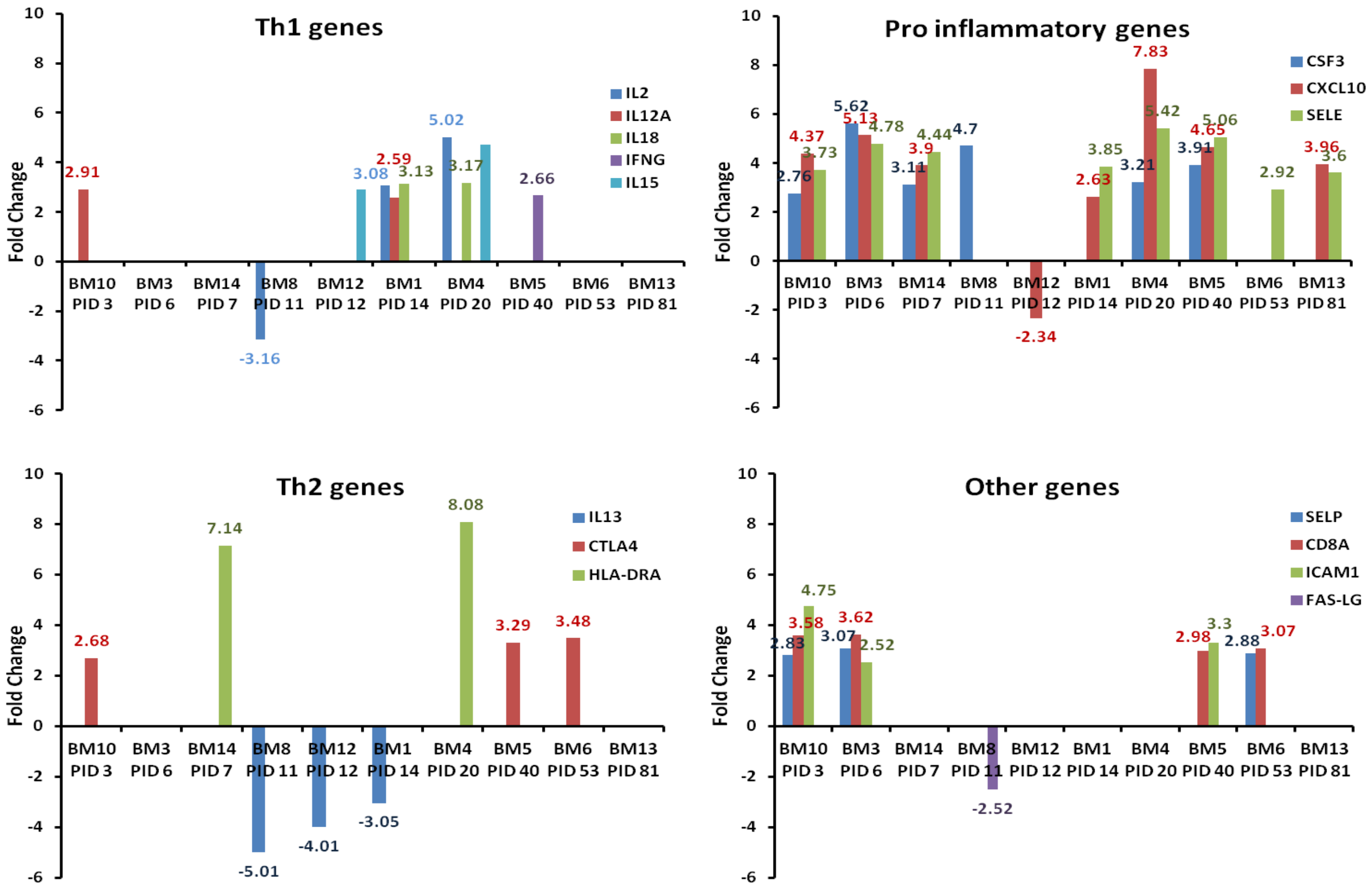

Supplementary Figure S1. Gene expression analysis. Fold changes observed in pro-inflammatory cytokines (A), Th1 genes (B), Th2 genes (C) and other genes (D) from total RNA extracted from spleen samples at the time of monkey sacrifice.
